# Central Nervous System (CNS) T-Cell Lymphoma as the Presenting Manifestation of Late-Onset Combined Immunodeficiency

**DOI:** 10.1155/2023/6650410

**Published:** 2023-10-18

**Authors:** Anthony Jeffrey, Luke A. Coyle, Dishan Samaranayake, Therese Boyle, James Drummond, Suran L. Fernando

**Affiliations:** ^1^Department of Haematology, Royal North Shore Hospital, St Leonards, NSW, Australia; ^2^Department of Medicine, The University of Sydney, Sydney, NSW, Australia; ^3^Department of Clinical Immunology and Allergy, Royal North Shore Hospital, St Leonards, NSW, Australia; ^4^Immunology Laboratory, Royal North Shore Hospital, New South Wales Health Pathology, St Leonards, NSW, Australia; ^5^Department of Radiology, Royal North Shore Hospital, Sydney, Australia

## Abstract

Late-onset combined immunodeficiency (LOCID), considered now a subset of common variable immunodeficiency (CVID) disorders, is characterized by a predominantly T-cell immune defect. LOCID has a distinct phenotype from CVID with a greater risk of lymphoproliferative complications. As compared to the CVID cohort, LOCID patients also have increased rates of splenomegaly and granulomatous disease. We report a case of central nervous system (CNS) T-cell lymphoma in a 67-year-old male as the presenting manifestation of LOCID. The patient achieved a complete response to therapy after 4 cycles of MATRix (methotrexate, cytarabine, and thiotepa) and 2 cycles of ICE (etoposide, carboplatin, and ifosfamide) chemotherapy followed by CNS-directed autologous stem cell transplantation. Intravenous immunoglobulin replacement was commenced to address the underlying immunodeficiency. Pulmonary lesions consistent with a diagnosis of granulomatous and lymphocytic interstitial lung disease (GLILD) were identified as a second noninfectious complication of LOCID. The pulmonary lesions resolved after chemotherapy and immunoglobulin replacement. The patient remains well with no evidence of disease recurrence now more than 18 months after completion of therapy. This is the first reported case of T-cell lymphoma in an adult patient with LOCID. Further study is needed to elucidate the mechanisms of transformation of B- or T-cells to lymphoproliferation in primary immunodeficiency patients as well as research to inform evidence-based therapeutic strategies for this challenging cohort of patients.

## 1. Introduction

Late-onset combined immunodeficiency (LOCID), considered now a subset of common variable immunodeficiency (CVID) disorders, is characterized by a predominantly T-cell defect with a CD4 count <200 cells/*μ*L and/or occurrence of an opportunistic infection. These patients have a high incidence of lymphoproliferative disease. We present the first reported case of T-cell lymphoma in LOCID in an adult.

## 2. Case Presentation

**Figure 1:**
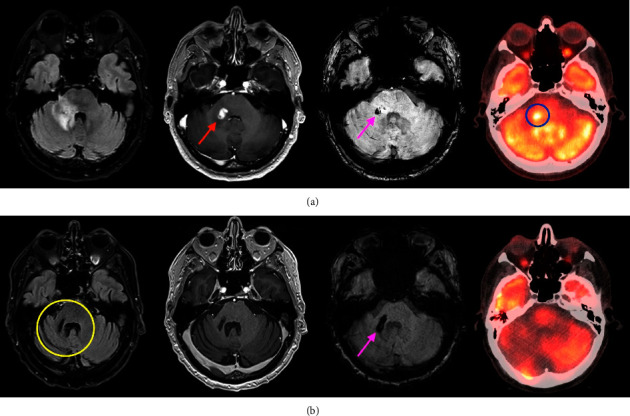
MRI and FDG-PET/CT at initial presentation and following treatment. At presentation (a) demonstrates a region of T2 FLAIR hyperintensity in the right middle cerebellar peduncle with an avidly enhancing 13 mm nodule (red arrow) on postcontrast T1. There was evidence of a small focus of intralesional haemorrhage on susceptibility weighted imaging (pink arrow). The lesion demonstrates moderately avid tracer uptake SUVMax 7.6 (blue ring). Following biopsy, chemotherapy, and ASCT (b), there was complete resolution of the T2 FLAIR signal (yellow ring); no residual enhancement on postcontrast T1 and haemosiderin staining (pink arrow). No residual FDG tracer uptake within this focus on FDG-PET.

A 67-year-old male presented to the emergency department with a 4-week history of dizziness, dysesthesia, and ataxia. His past medical history includes hypertension and melanoma in situ excised 2 months prior to presentation. Neurological examination demonstrated mild gait ataxia, subtle right-sided dysmetria, and saccadic intrusion of ocular pursuit movements in addition to right facial numbness in the V1 trigeminal nerve distribution. Cardiorespiratory and gastrointestinal examinations were unremarkable, and there was no evidence of lymphadenopathy or hepatosplenomegaly. Magnetic resonance imaging (MRI) of the brain demonstrated an enhancing lesion centred on the brachium pontis (13 × 10 × 10 mm) (Figure 1). Computed tomography (CT) scan of the chest, abdomen, and pelvis demonstrated multiple pulmonary nodular opacities with consolidation, ground glass change, and cavitation. On CT/fluorodeoxyglucose (FDG) positron emission tomography (PET), the lung lesions were not FDG-avid; however, the brainstem lesion was mildly FDG-avid (Figure 1). Cerebrospinal fluid (CSF) analysis was unremarkable including tests for cytology, microbiology, flow cytometry, and oligoclonal bands. Due to the risks of brain biopsy, the lung lesions were biopsied demonstrating non-necrotising granulomatous inflammation with mostly CD3+ lymphocytes, with no evidence of malignancy on cytology. Methenamine silver staining highlighted short septate fungal hyphae with changes in organizing pneumonia at the periphery. The microscopic appearance was insufficient for definitive fungal speciation; however, principal differentials included *Aspergillus* and *Fusarium* species. *Aspergillus* and pan-fungal PCR and culture were negative.

Stereotactic brain biopsy was subsequently performed due to clinical and radiological progression despite 10 weeks of antifungal therapy involving 300 mg oral voriconazole twice daily with liposomal amphotericin 3 mg/kg IV once daily administered concurrently for the initial 6 weeks of therapy. An extended trial of antifungal therapy was deemed necessary to adequately assess response and test the hypothesis that systemic fungal infection represented the unifying diagnosis for the cerebellar mass and lung opacities. Despite acknowledging the diagnostic uncertainty inherent in this approach, the decision was made in the context of the high likelihood of permanent neurological deficits from biopsy of the cerebellar lesion. Histopathology demonstrated an atypical lymphohistiocytic angioinvasive and angiodestructive inflammatory infiltrate with loosely formed granulomas surrounding occasional foci of punctate necrosis. The majority of the lymphocytes were CD3 positive, most T-cells expressed CD4 and BF-1, and there was increased granzyme staining. The Ki67 proliferative index was 50–60%, higher than would be expected for a reactive population. The degree of nuclear atypia was also not compatible with a reactive process or vasculitis. Epstein–Barr virus (EBV)-encoded RNA in situ hybridisation (EBER-ISH) was negative, therefore excluding lymphomatoid granulomatosis. No organisms were seen on periodic acid Schiff stain, methenamine silver stain, Ziehl–Neelson stain, or Fite stain. Toxoplasma antigen was negative. Independent and blinded review of the histopathology was undertaken at 2 tertiary institutions with histiocyte-rich T-cell lymphoma with a florid granulomatous response reported as the leading differential diagnosis. The histopathological findings were considered to be most consistent with a WHO diagnosis of peripheral T-cell lymphoma, NOS. Bone marrow biopsy did not show involvement with T-cell lymphoma.

Initial investigations also revealed panhypogammaglobulinemia IgG 2.6 g/L (7.0–14), IgA 0.42 g/L (0.7–4.0), and IgM 0.24 g/L (0.4–2.3) with T- and B-cell cell lymphopenia (CD4+184 cells/*μ*L, CD8+50 cells/*μ*L, and CD19+170 cells/*μ*L) with normal numbers of CD16+/56+ cells in the absence of a history of infectious complications. Further investigations for immunodeficiency revealed negative human immunodeficiency virus (HIV) serology and normal 24 hour urine and faecal protein studies. He had absent isohemagglutinins. Functional antibody responses were impaired, as measured before and after 23-valent antipneumococcal vaccine. The total antipneumococcal IgG measured 17.5 and 36.3 mg/mL, respectively (normal >2-fold increase and >110 mg/mL postvaccination). A diagnosis of late-onset combined immunodeficiency was made with the noninfectious complications of granulomatous and lymphocytic interstitial lung disease (GLILD) and T-cell lymphoma. Immunophenotyping revealed normal proportion of class-switched memory B-cells and CD21 low B-cells consistent with the CVID classifications of EuroClass B+/smB+/CD21-lo/Tr-lo, Paris phenotype MB2, and Freiburg phenotype 2. Massively parallel sequencing (Clinical Research Exome v2XX; Agilent, Santa Clara, California) detected c.2468T > C in the *TTC7A* gene resulting in an amino acid substitution at p.leu823pro. This variant has been reported in an individual with combined immunodeficiency and intestinal atresia [1] and in an individual with gastrointestinal defects and combined immunodeficiency syndrome (GIDID) [2].

Treatment was initiated with a combination chemotherapy regimen comprising of methotrexate 3500 mg/m^2^, cytarabine 2000 mg/m^2^ BD for 2 days, and thiotepa 30 mg/m^2^ (MATRix) administered in 21 day cycles [3]. Four cycles of MATRix chemotherapy were administered followed by two cycles of ICE (etoposide 100 mg/m^2^, carboplatin 5 AUC, and ifosfamide 5000 mg/m^2^). Intravenous immunoglobulin replacement was commenced with a maintenance dose of 0.4 g/kg administered monthly. Progress MRI demonstrated improvement in appearance of the cerebellar lesion with reduction in T2 FLAIR signal and reduction in peripheral contrast enhancement following chemotherapy. Resolution of pulmonary lesions was demonstrated on progress PET scan. The patient subsequently underwent central nervous system (CNS)-directed autologous haematopoietic stem cell transplant (auto-HSCT) with carmustine and thiotepa conditioning [4]. MRI of the brain was performed post-transplant demonstrating resolution of all enhancing CNS lesions consistent with a complete response (CR) (Figure 1).

### 2.1. Long-Term Follow-Up

The most recent gadolinium-enhanced MRI of the brain was performed 18 months post-autologous stem cell transplant demonstrating no evidence of residual/recurrent disease with an unchanged appearance of the treatment bed in the right middle cerebellar peduncle. Trough immunoglobulin levels demonstrate persistent hypogammaglobulinemia, and indefinite long-term immunoglobulin replacement has been recommended. The patient has persistent neurological deficits primarily manifesting as balance issues and right-sided incoordination. Neurological examination demonstrates a narrow-based gait and difficulty with tandem gait. There is mild finger-nose ataxia on the right and moderate heel-knee-shin ataxia on the right. Substantial improvements have been made in the patients' function with rehabilitation; however, given the cerebellar atrophy on MRI, it felt unlikely that he will return to his preillness functional status.

## 3. Discussion

LOCID has a distinct phenotype from CVID with a greater risk of lymphoproliferative complications. The French DEFI study showed that LOCID patients as compared to the CVID cohort had a higher prevalence of splenomegaly (64% vs. 31%), granulomatous disease (43% vs. 10%), and lymphoma (29% vs. 4%) [5]. T-cell lymphoma is rare as revealed in a systematic review on predisposition to B- and T-cell neoplasia in patients with inborn errors of immunity [6]. There is one previous report of stage IV T-cell lymphoma in a 8-year-old who died from sepsis 2 years after complete response with NHL-BFM 95 treatment [7]. CVID patients with lymphoproliferative disease as defined by lymphocytic inflammation (lymphadenopathy and lymphocytosis), organ- (lung and gastrointestinal) and nonorgan-specific lymphocytic inflammation, and monoclonal gammopathies appear at increased risk of lymphoma. The United States Immunodeficiency Network registry showed that patients with lymphoproliferative disease were more likely to have a diagnosis of lymphoma (8%) compared with CVID patients without other lymphoproliferative conditions (OR = 2.5; *p* = 0.005) [8]. Our patient with GLILD may have been at inherent risk but precise mechanisms that predispose to transformation of B- or T-cells to lymphoproliferation and/or development of lymphomas in patient with primary immunodeficiency disorders requires further elucidation. An intrinsic susceptibility to DNA damage may provide conditions for neoplastic transformation, impaired apoptosis of damaged cells, and premature cellular senescence. Oncogenic viruses such as EBV, human herpesvirus-6 (HHV6), human herpesvirus-8 (HHV8), and human papillomavirus (HPV) may further proliferate adaptive lymphocytes in the context of an immunodeficiency [9].

The treatment of CNS T-cell lymphoma in the context of CVID presents numerous therapeutic challenges. Central nervous system T-cell lymphoma is rare, even in the immunocompetent population, with 98% of primary or secondary CNS lymphomas being of B-cell origin [10]. Furthermore, the majority of published data evaluating the treatment of CNS lymphoma in immunocompromised individuals are in the context of HIV infection and post-transplant lymphoproliferative disorders which are almost exclusively EBV-driven and also of B-cell origin [11]. In immunocompetent individuals, current guidelines recommend a similar treatment approach to T-cell CNS lymphoma to CNS lymphoma of B-cell origin, i.e., high dose methotrexate-based combination chemotherapy regimens with either whole brain radiotherapy or auto-HSCT consolidation. As such, treatments for T-cell lymphoma in CVID disorders, particular with CNS involvement, are poorly defined with treatment based on expert opinion and extrapolated from the immunocompetent population; however, limited studies have demonstrated an inferior response to chemotherapy and an associated higher susceptibility to infectious complications in this patient population [6]. Our case raises awareness of T-cell lymphoma as a manifestation of CVID disorders including LOCID and the need for further study in order to inform evidence-based therapeutic strategies for this challenging cohort of patients.

## Data Availability

Data were not generated for this case report. Additional information regarding the case can be requested from the corresponding author.
